# Early exposure to secondhand tobacco smoke and the development of allergic diseases in 4 year old children in Malmö, Sweden

**DOI:** 10.1186/1471-2431-10-61

**Published:** 2010-08-23

**Authors:** Kristina Hansen, Elisabeth Mangrio, Martin Lindström, Maria Rosvall

**Affiliations:** 1 Scania University Hospital, Malmö, Sweden; 2 Department of Clinical Sciences, Lund University, Sweden

## Abstract

**Background:**

Earlier studies have shown an association between secondhand tobacco smoke and allergy development in children. Furthermore, there is an increased risk of developing an allergy if the parents have an allergy. However, there are only few studies investigating the potential synergistic effect of secondhand tobacco smoke and allergic heredity on the development of an allergy.

**Methods:**

The study was population-based cross-sectional with retrospective information on presence of secondhand tobacco smoke during early life. The study population consisted of children who visited the Child Health Care (CHC) centres in Malmö for their 4-year health checkup during 2006-2008 and whose parents answered a self-administered questionnaire (n = 4,278 children). The questionnaire was distributed to parents of children registered with the CHC and invited for the 4-year checkup during the study period.

**Results:**

There was a two to four times increased odds of the child having an allergy or having sought medical care due to allergic symptoms if at least one parent had an allergy, while there were rather small increased odds related to presence of secondhand smoke during the child's first month in life or at the age of 8 months. However, children with heredity for allergies and with presence of secondhand tobacco smoke during their first year in life had highly increased odds of developing an allergy and having sought medical care due to allergic symptoms at 4 years of age. Thus, there was a synergistic effect enhancing the independent effects of heredity and exposure to secondhand tobacco smoke on allergy development.

**Conclusions:**

Children with a family history of allergies and early exposure to secondhand tobacco smoke is a risk group that prevention and intervention should pay extra attention to. The tobacco smoke effect on children is an essential and urgent question considering it not being self chosen, possibly giving life lasting negative health effects and being possible to reduce.

## Background

Today about 1 billion people smoke worldwide and every second child is exposed to secondhand tobacco smoke [1]. In Sweden about 1 million people smoke, 11% of the men, 14% of the women [2] and 6% of the pregnant women [3]. Even though secondhand tobacco smoke has decreased during later years, about 6% of Swedish mothers smoked when the child was 0 to 4 weeks and 7% when the child was 8 months old, while 11% of the fathers smoked when the child was 8 months of age during 2006 [3]. Exposure to secondhand tobacco smoke is today considered a risk factor for the development of lower respiratory illness among young children [4], and secondhand tobacco smoke has been shown to be associated with asthma and wheezing episodes until 6 years of age [5], allergic sensitization and sensitization to food allergens [6,7], and the development of atopic eczema [8]. Furthermore, among children with an ongoing asthma, secondhand tobacco smoke exposure is considered to cause a more severe course [5]. However, research considering exposure to secondhand tobacco smoke and allergic sensitization present inconclusive results [9]. Furthermore, while earlier studies have shown an increased risk of developing an allergy if the parents have an allergy, there are only few studies [8,10-12] investigating the potential synergistic effect of secondhand tobacco smoke and heredity on the development of an allergy. For example, in the study by Krämer et al. there was an association between cotinine to creatinine ratio (CCR), as a measurement of ETS, and sensitization against house dust mites among children with parental atopy [8].

The aim of the present study was to investigate the association between secondhand tobacco smoke at an early age and presence of allergy/having sought care due to allergic symptoms in 4 year old children in Malmö, the biggest city in the county of Scania, Sweden. We also wanted to examine whether a potential association between secondhand tobacco smoke was similar in children with heredity for allergy, i.e., with at least one parent having an allergy and children with no such heredity.

## Methods

### Study population

This study was conducted in Malmö, the third largest city in Sweden. It is a multiethnic city, where 27% of the inhabitants being of foreign origin, and every other child born having at least one parent born in another country. The study was population-based cross-sectional with retrospective information on presence of secondhand tobacco smoke during early life. The study population was 4 year old children from Malmö, who visited the Child Health Care (CHC) centres for their 4-year checkup during 2006-2008 and whose parents answered a self-administered questionnaire (n = 4,278 children), i.e, 67% of the children who received the questionnaire. The CHC centres in Sweden are a well-established organisation with a strong tradition, monitoring children's physical and developmental health in order to reduce mortality, morbidity and disability among new born and young children. The CHC centres offer base programs including regular visits, controlling each child's weight, length, hearing, sight, physical and psychological development and administration of vaccinations according to the base program, until the child is 5-6 years old. The base program at the CHC centres includes 15 visits from age 0 to age 4 years. Some of the visits are conducted with a nurse and some of the visits with a nurse and a psysician. The CHC centres support and educate parents concerning childcare and child development. The emphasis is prevention of ill-health, the consultations are free of charge and optional. The CHC focus is prevention; visits are voluntary and the consultations are free of charge. As many as 99% of children aged 0-6 participate in the programme [13].

The questionnaire addresses issues such as the child's family situation, parents' educational level, country of birth, occupation and emotional and financial security. It also covers questions about weight, allergies, maternal smoking during pregnancy, secondhand tobacco smoke, breastfeeding and antibiotic consumption. The questionnaire was validated and tested for reliability, and translated into five different languages: Albanian, Arabic, English, Serbo-Croatian, and Somali [14].

### Allergy definition

*Having an allergy *was assessed through parents reporting that the child had or had had allergic diseases (i.e., atopic eczema, hay fever, asthma or food allergies). Nickel and penicillin allergies were not considered. The categories were; no allergies, suspected allergies, confirmed allergy tested positive in skin prick test, in blood test or by provocation, or severe allergy diagnosed by a physician with need of medication for at least three months of the year, and the last category was I don't know. Those reporting confirmed allergies or having severe allergies were considered as having an allergy. *Parental allergy *was assessed through an identical question, but directed at each biological parent instead. Parents reporting confirmed allergies or having severe allergies were considered as having an allergy. *Presence of allergy *was further assessed by the question if the child had sought medical care in addition to the regular CHC-visits during the last 12 months, due to atopic eczema, food allergies or asthma.

### Secondhand tobacco smoke and smoking during pregnancy

*Secondhand tobacco smoke during early life *was assessed by the question: Did anyone in the family smoke when the child was 0-4 weeks of age? The answering alternatives were: No, yes - mother/stepmother smoked on a daily basis (also including outdoor smoking), or yes-father/stepfather smoked on a daily basis (including outdoor smoking), or yes-siblings or other person smoked on a daily basis (including outdoor smoking). *Secondhand smoking at 0-4 weeks of age *was divided into no (no secondhand smoking at all) and yes (daily secondhand tobacco smoke, including smoking outside). An identical question was used to assess *secondhand smoking at 8 months of age *and a similar question was also used to assess secondhand tobacco smoke at 4 years of age. *Maternal smoking during pregnancy *was divided into yes and no.

### Parental and child characteristics

*Parents' country of birth *was divided into: both parents born in Sweden, one parent born in Sweden, and both parents born outside Sweden. *Maternal educational level *was based on years of schooling and divided into 9 years and less, 10-12 years, and more than 12 years of education. *Taken part of parental education program *was divided into taken part or not taken part. *Crowded living *was defined as a household having more than 2 persons per room excluding the kitchen and toilet. *Emotional support *was assessed with the question: Do you have someone that can give you proper personal support to cope with life's stress and problems? with answers being classified into high emotional support (definitely yes or probably yes) and low emotional support (not for certain or no).

Characteristics of the child were also assessed. *Low birth weight *was classified as <2500 g, and *normal birth weight *as ≥2500 g. *Position among siblings in the family *was dichotomised into firstborn versus secondborn or later. *Having been breastfed *was classified into: yes or no. *Having a pet *was divided into yes or no.

### Statistical methods

Logistic regression was used to analyse the associations between secondhand tobacco smoke and the development of an allergy. Multiple logistic regression analyses were performed in order to adjust the estimated odds ratios (OR) for the influence of potential confounding factors, i.e., sex, year of investigation, (i.e., 2006, 2007 or 2008), maternal smoking during pregnancy, maternal educational level, parents' country of birth, taken part of parental education program, crowded living and having a pet. Furthermore, stratified analyses were performed with regard to secondhand tobacco smoke in relation to the heredity for allergy. Potential synergistic effects between presence of secondhand smoke and presence of parental allergy were tested using logistic regression analysis, adjusted for potential confounders. Synergi index = ((OR(AB) - 1)/(OR(Ab) - 1) + (OR(aB) - 1)) where OR = Odds ratio; Ab = exposed to one risk factor; aB = exposed to the other risk factor; AB = exposed to both risk factors. Synergistic interaction was defined to be present if the effect of both exposures was more than additive compared with their independent effects (synergy index, S > 1) [15]. Confidence intervals (95%) for the synergy indexes were calculated [16]. Statistical analyses were performed with version 17.0 of SPSS for Windows. The study was approved by the Regional Ethical committee, Lund University.

## Results

Table 1 describes the associations between characteristics of 4-year old children and presence of secondhand tobacco smoke early in life. In total, 894 (22%) of the 4-year-old children had presence of secondhand tobacco smoke during their first month in life. Children with such presence of secondhand smoke more often had a mother with low educational level, had parents not born in Sweden, had a mother who smoked during pregnancy, had a pet, had crowded living, had less often taken part in parental educational programs, less often had a parent with an allergy, had less often been breastfed and were less often firstborn compared to children not exposed to secondhand smoking. There were no statistically significant differences with regard to sex, low birth weight and low emotional support. Children with presence of secondhand tobacco smoke at 0-4 weeks of age also to a very high degree had presence of secondhand tobacco smoke at 8 months and at 4 years of age. A similar pattern of association was seen for presence of secondhand smoking at 8-months of age. Most of the factors used as confounders in the forthcoming analyses showed associations both with presence of secondhand tobacco smoke and presence of allergies/having sought medical care due to allergic symptoms at 4 years of age.

**Table 1:** Characteristics of 4-year old children by presence of secondhand tobacco smoke during early life, in Malmö, Sweden, 2006-2008

	Secondhand tobacco smoke during the first month in life*		Secondhand tobacco smoke at 8 months of age*	
	No	Yes	No	Yes
	(n = 3211; 78%)	(n = 894; 22%)	(n = 2968; 76%)	(n = 925; 24%)
*Sociodemographic characteristics*				
Male (%)	49.7	50.2	49.3	49.5
Firstborn (%)	46.7	42.7†	47.2	44.6
Mother having a low educational level (%)	9.8	21.2†	8.7	21.0†
Both parents born outside Sweden (%)	26.0	39.1†	24.2	38.9†
At least one parent with an allergy (%)	39.8	32.0†	39.8	32.0†
Crowded living (%)	9.4	14.5†	8.8	14.1†
*Early life factors*				
Secondhand tobacco smoke at 1 month* (%)			1.5	89.8†
Low birth weight (<2500 g) (%)	6.0	6.5	5.9	6.5
Mother smoking during pregnancy (%)	2.5	35.3†	1.9	35.3†
Secondhand tobacco smoke at 8 months* (%)	3.1	94.9†		
No breastfeeding (%)	4.4	6.8†	4.5	6.9†
Taken part of parental education (%)	57.8	44.1†	58.5	45.2†
*Environmental factors at 4 years*				
Secondhand tobacco smoke at 4 years* (%)	7.7	71.9†	6.0	73.6†
Having a pet (%)	22.0	29.8†	22.5	29.4†
Low emotional support (%)	19.1	23.4	18.7	22.0

*Secondhand tobacco smoke at 0-4 weeks and 8 months of age, respectively, was categorized into no (no secondhand tobacco smoke at all) and yes (daily secondhand tobacco smoke, including smoking outside).

† Statistically significantly (p < 0.05) different compared those not exposed to secondhand tobacco smoke at the same age.

Table 2 shows the adjusted associations between early secondhand tobacco smoke and parental allergy on the one hand, and presence of allergy at four years of age and having sought care due to allergic symptoms, respectively, on the other hand. Adjustments were made for potential confounders. There was a two to four times increased odds of the child having an allergy or having sought medical care due to allergic symptoms if at least one parent had an allergy, while there were rather small increased odds related to presence of secondhand smoke during the child's first month in life or at the age of 8 months.

**Table 2:** Odds ratios (OR) and 95% confidence intervals (CI) of having an allergy or having sought medical care due to allergic symptoms during the last year in 4-year old children in Malmö, Sweden, by presence of secondhand tobacco smoke and presence of parental allergy

	Allergy†	Having sought medical care† due to allergic symptoms
	
	Adjusted model‡ OR; 95% CI*	Adjusted model‡ OR; 95% CI*
*Secondhand tobacco smoke*		
Secondhand tobacco smoke during the first month of age (yes vs. no)*	1.20 (0.83, 1.73)	1.17 (0.81, 1.69)
Secondhand tobacco smoke during eight month of age (yes vs. no)	1.12 (0.77, 1.64)	1.39 (0.97, 1.99)
*Parents with an allergy*		
At least one parent with an allergy (yes vs. no)	4.79 (3.51, 6.71)	2.46 (1.81, 3.34)
Mother having an allergy (yes vs. no)	4.85 (3.58, 6.56)	2.57 (1.89, 3.49)
Father having an allergy (yes vs. no)	3.51 (2.61, 5.01)	1.74 (1.25, 2.41)

* OR, odds ratio; CI, confience interval; Secondhand tobacco smoke was categorized into no (no secondhand tobacco smoke at all) and yes (daily secondhand tobacco smoke, including smoking outside).

† Parents reporting that the child had or had had allergic diseases (i.e., atopic eczema, hay fever, asthma or food allergies). Those reporting confirmed allergies (tested positive in skin prick test, in blood test and by provocation) or having severe allergies were considered as having an allergy;. Allergies were further assessed by the question if the child had sought medical care during the last 12 months, due to atopic eczema, food allergies or asthma.

‡ Adjusted for sex, year, maternal smoking during pregnancy, maternal educational level, parents'country of birth, taken part of parental education program, crowded living and having a pet.

There was complete information on the children having or having had an allergy, presence of an allergy among the parents and presence of secondhand tobacco smoke in 2860 children (for secondhand smoke at 0-4 weeks) and 2740 children (for secondhand smoke at 8 months) (i.e., two thirds of the study population). Table 3 presents four groups constructed based on presence of secondhand tobacco smoke early in life and presence of parental allergy, i.e., children with no presence of daily secondhand tobacco smoke during early life and whose parents had no allergy (reference group), children with presence of daily secondhand tobacco smoke during early life and whose parents had no allergy, children with no presence of daily secondhand tobacco smoke during early life and with at least one parent having an allergy and children with presence of daily secondhand tobacco smoke during early life and with at least one parent having an allergy. Children with heredity for allergies and with presence of secondhand tobacco smoke during their first month in life had highly increased odds of developing an allergy, while no such effect was seen among children with presence of secondhand tobacco smoke but without heredity for allergies. The synergy index was 2.05 (95% CI: 1.09, 3.86). As it is larger than 1 it shows a synergistic effect of parental allergy and presence of secondhand smoke during the first month in life on developing an allergy up to the age of four years. A similar pattern of association was seen for presence of secondhand smoke at 8 months of age (table 3) and with a synergy index of 1.48 (95% CI: 0.79, 2.79) (data not shown in table).

**Table 3:** Adjusted odds ratios (OR) and 95% confidence intervals (CI) of having or having had an allergy by presence of secondhand tobacco smoke early in life and presence of parental allergy in 4-year old children, Malmö, Sweden

*No parent with an allergy*		*At least one parent with an allergy*	
	
Secondhand tobacco smoke at 0-4 weeks of age*		Secondhand tobacco smoke at 0-4 weeks of age*	
	
No	Yes	No	Yes
OR; 95% CI*	OR; 95% CI*	OR; 95% CI*	OR; 95% CI*
	
1.00†	0.96 (0.50, 1.81)§	4.31 (3.07, 6.97)§	7.69 (4.46, 13.25)§¶
	
Secondhand tobacco smoke at 8 months of age*		Secondhand tobacco smoke at 8 months of age*	
	
No	Yes	No	Yes
	
OR; 95% CI*	OR; 95% CI*	OR; 95% CI*	OR; 95% CI*
	
1.00†	1.15 (0.63, 2.07)§	4.34 (3.06, 6.17)§	6.10 (3.51, 10.59)§

* OR, odds ratio; CI, confidence interval; Secondhand tobacco smoke at 0-4 weeks and 8 months were categorized into no (no secondhand tobacco smoke at all) and yes (daily secondhand tobacco smoke, including smoking outside).

† Reference level.

‡ Parents reporting that the child had or have had allergic diseases (i.e., atopic eczema, hay fever, asthma or food allergies). Those reporting confirmed allergies (tested positive in skin prick test, in blood test and by provocation) or having severe allergies were considered as having an allergy.

§ Adjusted for sex, year, maternal smoking during pregnancy, maternal educational level, parents' country of birth, taken part of parental education program, crowded living and having a pet.

¶ Statistically significantly different (p < 0.05) from the category: At least one parent with an allergy and no secondhand tobacco smoke.

There was complete information on having sought medical care due to allergic symptoms, presence of an allergy among the parents and presence of secondhand smoke in 3264 children (for secondhand smoke at 0-4 weeks) and 3136 children (for secondhand smoke at 8 months) (i.e., three quarters of the study population). Table 4 presents the odds of having sought medical care due to allergic symptoms during the last year using the same categorization as mentioned above. Children with heredity for allergies and with presence of secondhand tobacco smoke during their first month had highly increased odds of having sought medical care due to allergic symptoms, while no such effect was seen among children with presence of secondhand tobacco smoke but without heredity for allergies. The synergy index was 2.24 (95% CI: 0.94, 5.34). As it is larger than 1 it indicates a synergistic effect of parental allergy and presence of secondhand smoke during the first month in life on having sought medical care due to allergic symptoms during the last year at 4 years of age. However, the synergy index was not statistically significant. A similar and somewhat stronger association was seen for secondhand smoke at 8 months of age (table 4) and with a synergy index of 3.26 (95% CI: 1.33, 8.00)) (data not shown in table).

**Table 4:** Adjusted odds ratios (OR) and 95% confidence intervals (CI) of having sought medical care due to allergic symptoms during the last year by presence of secondhand tobacco smoke early in life and presence of parental allergy in 4-year old children, Malmö, Sweden

*No parent with an allergy*		*At least one parent with an allergy*	
	
Secondhand tobacco smoke at 0-4 weeks of age*		Secondhand tobacco smoke at 0-4 weeks of age*	
	
No	Yes	No	Yes
OR; 95% CI*	OR; 95% CI	OR; 95% CI	OR; 95% CI
	
1.00†	0.97 (0.53, 1.77)§	2.26 (1.61, 3.18)§	3.77 (2.20, 6.44)§
	
Secondhand tobacco smoke at 8 months of age*		Secondhand tobacco smoke at 8 months of age*	
	
No	Yes	No	Yes
	
OR; 95% CI*	OR; 95% CI	OR; 95% CI	OR; 95% CI
	
1.00†	1.04 (0.58, 1.86)§	2.02 (1.42, 2.87)§	4.46 (2.68, 6.44)§¶

* OR, odds ratio; CI, confience interval; Secondhand tobacco smoke at 0-4 weeks and 8 months were categorized into no (no secondhand tobacco smoke at all) and yes (daily secondhand tobacco smoke, including smoking outside).

† Reference level.

‡ Presence of allergy was assessed by the question if the child had sought medical care during the last 12 months, due to atopic eczema, food allergies or asthma.

§ Adjusted for sex, year, maternal smoking during pregnancy, maternal educational level, parents'country of birth, taken part of parental education program, and having a pet.

¶ Statistically significantly different (p < 0.05) from the category: At least one parent with an allergy and no secondhand tobacco smoke.

## Discussion

This population-based study on 4-year old children in Malmö, Scania, Sweden, showed that presence of secondhand tobacco smoke at an early age tended to be associated with a somewhat increased prevalence of reported allergies and allergic symptoms although these associations were non-significant. However, children with heredity for allergies and with presence of secondhand tobacco smoke during early life had highly increased odds of developing an allergy and having sought medical care due to allergic symptoms.

Early exposure to secondhand smoking has been shown to be associated with the development of asthma and wheezing episodes [5], allergic sensitization and sensitization to food allergens [6,7], and the development of atopic eczema [8]. If established asthma is present, secondhand tobacco smoke is associated with a more severe course [5]. However, there are also studies showing no associations between exposure to secondhand tobacco smoke and allergic sensitization [9] and a larger effect of prenatal than postnatal exposure of tobacco on sensitization in children [12]. Furthermore, as in this study, earlier studies have shown an increased risk of developing an allergy if the parents have an allergy (8,10-12). This is in consensus with established theories of allergy heredity and also confirmed by the Swedish BAMSE study, a prospective birth cohort study among children (1-14 years of age) in four areas in Stockholm started in 1994. The study concludes that there is a 24% risk for future asthma and allergies if the child's parents don't have allergies or asthma and a 55% risk if both parents are allergic [17].

Assessment of interaction is often performed by simply introducing product terms into logistic risk models. This practice has been criticized with arguments that the product term expresses interaction in a statistical meaning and not in a biological sense [18]. Rothman's model of synergism used in the present study is based on the theory of two causes being component causes in the same sufficient cause [19]. The criterion for interaction is departure from additivity and the reference group for the comparisons is the group unexposed to either factor. Rothman's model of synergism is based on additive models and was recommended in a review on how to evaluate interaction by Hallqvist et al. [19]. This model has the advantages in that information from different types of studies can be employed, it has a theoretical background, and there is methodology to obtain confidence intervals from multiple regression software [19]. There are also other methods of assessing measures of interaction as departure from additivity such as the attributable proportion (AP) and the relative excess risk due to interaction (RERI) [20]. In the present study, early exposure to secondhand tobacco smoke and having allergic parents seemed to have a synergistic effect on the development of an allergy and allergic symptoms, enhancing the independent effects of heredity and secondhand tobacco smoke. Similar results were found in a study by Jaakkola et al. using a similar methodology of measuring interaction concluding that genetic heritage is a factor affected by secondhand smoke conditions and the odds of having asthma was more than twofold in children with an atopic heredity and early presence of secondhand tobacco smoke [10]. Similar results were also found in a prospective multicenter birth cohort study by Keil et al. showing an increased risk of allergic sensitization and the development of asthma symptoms if the mother was smoking and if having allergic parents, while there was no increased risk if only having a smoking mother but no heredity [11]. Furthermore, several experimental genetic studies confirm that genetic factors and secondhand factors act together and might modulate the development of allergy and asthma [21-24].

We found that parents not taken part of parental education more often exposed their children to secondhand smoke than parents taken part. Other studies show that there is a value in parental education in different forms, and it may lead to decreased exposure of the child [25,26]. Furthermore, in our study children with parents having an allergy were exposed to tobacco smoke to a lower degree than children with parents without allergies. Similar results have been shown in other studies [27].

Certain methodological issues need to be addressed. Parents with 4-year-old children were at the Child Health Care centres visit at 4-years of age (99% of children aged 0-6 participate in the Child Health Care centres programme) [13] invited to participate in the study by answering the questionnaire. The total number of children whose parents answered the questionnaire during the study period was 67% or about two thirds of all those who received the questionnaire. An earlier study showed only small differences between participants and non-participants with regard to being born in Sweden or not and the parents' educational level [14]. Self-reported smoking is a potential source of bias. A recent review found a tendency of underestimation of self-reported smoking, especially when smoking is seen as undesirable i.e., during pregnancy [28]. Such an underestimation would lead to a dilution of the true associations and the results might be even stronger if corrected for this underestimation. However, there are also studies that have validated questionnaire information on presence of secondhand smoke in early childhood through blood samples measuring cotinine and thiocyanate in serum with good agreement between high and low levels of biomarkers and daily and non-smoking mothers [29]. Furthermore, a Danish study from 2004 showed a high validity of self-reported smoking among parents during pregnancy and early childhood. Repeated interviews and CO measurements in a prospective study design did not change the validity, indicating a low risk of information bias [30]. Moreover, in our study, children exposed to secondhand tobacco smoke at 0 to 4 weeks were also to a very high degree exposed at 8 months and still highly exposed at 4 years of age. This suggests a consistency in the assessments and that the same children are exposed over a long period of time. Self-reported allergies might be another potential source of bias. Parents who suffer from allergies or who smoke may be more alert in seeking medical care for their children based on recognizing symptoms. The questions used to code the children as having an allergy include allergy confirmed through skin prick test, blood sample, or by provocation, or having it diagnosed by a physician and requires medication at least three months of the year or having sought medical care due to allergic symptoms care in addition to the regular CHC-visits. These groups have rather severe allergic problems and we believe that the risk for inaccurate reporting should be rather small. Torén et al confirms that self reported asthma is more accurate in severe cases than among mild cases [31]. In all, 87% of those with clinically diagnosed asthma in adult age reported that they had or had ever had asthma and where those with moderate or severe asthma were 6 times more prone to report having or having had asthma compared to the mild cases [31]. One weakness of our study is that we only have complete information on secondhand smoke, parental as well as child allergies in about two thirds of the population. Children with such missing information were similar to the whole study population with regard to sex, but more often had low educated mothers and more often had presence of secondhand smoke at 0-4 weeks and at 8 months in life than children with complete data. On those children with information on either allergy in the child or parental allergy, the prevalences of both child and parental allergies were higher than among those included in the analyses. Since we do not have information on the prevalence of child allergy among those with missing data in more than part of the population it is hard to say anything about the effect of the missing data on the found associations. One strength of the present study was the fact that the questionnaire had been tested for reliability and translated into five different languages [14]. We chose to adjust for the following confounding factors: sex, year, maternal smoking during pregnancy, maternal educational level, parents' country of birth, taken part of parental education, crowded living and having a pet. However, there might have been other confounding factors not included in our models.

## Conclusion

This study shows that while presence of secondhand tobacco smoke at an early age tended to be associated with a somewhat increased prevalence of reported allergies and allergic symptoms, children with heredity for allergies and with presence of secondhand tobacco smoke during early life had highly increased odds of developing an allergy. Thus, there was a synergistic effect enhancing the independent effects of heredity and exposure to secondhand tobacco smoke on allergy development. The secondhand tobacco smoke effects on children is an essential and urgent question considering it not being self chosen, possibly giving life lasting negative health effects and being possible to reduce. Children with both a family history of allergies and exposure to secondhand tobacco smoke is a risk group that prevention and intervention should pay extra attention to.

## Competing interests

The authors declare that they have no competing interests.

## Authors' contributions

KH and MR have contributed to the conception of the work, the analysis of the data, the interpretation and the discussion of the results, the drafting, writing, and revision of the content. EM has contributed to the interpretation and the discussion of the results, the writing, and revision of the content. ML has contributed to the interpretation and the discussion of the results. All authors read and approved the final manuscript.

## Pre-publication history

The pre-publication history for this paper can be accessed here:

http://www.biomedcentral.com/1471-2431/10/61/prepub

## References

[B1] WHO Tobacco key facts http://www.who.int/topics/tobacco/facts/en/index.html

[B2] The Swedish National Institute of Public Health Hälsa på lika villkor http://www.fhi.se/Documents/Statistik-uppfoljning/Folkhalsoenkaten/Resultat-2008/Halsa-pa-lika-villkor-2008.pdf (In Swedish)

[B3] National Board of Health and Welfare Public Health Report 2009: Barns hälsa 2009 Stockholm (In Swedish)

[B4] Strachan DP Cook DG Health effects of passive smoking 1, Parental smoking and lower respiratory illness in infancy and early childhood Thorax 1997 52 905 914 10.1136/thx.52.10.905 9404380 PMC1758431

[B5] Strachan DP Cook DG Health effects of passive smoking 6, Parental smoking and childhood asthma: longitudinal and case control studies Thorax 1998 53 204 212 10.1136/thx.53.3.204 9659358 PMC1745164

[B6] Lannerö E Wickman M van Hage M Bergström A Pershagen G Nordvall L Exposure to environmental tobacco smoke and sensitisation in children Thorax 2008 63 172 176 10.1136/thx.2007.079053 18089631

[B7] Kulig M Luck W Lau S Niggemann B Bergmann R Klettke U Guggenmoos-Holzmann I Wahn U Effect of pre-and postnatal tobacco smoke exposure on specific sensitization to food and inhalant allergens during the first 3 years of life Allergy 1999 54 Multicenter Allergy Study Group, Germany 220 228 10.1034/j.1398-9995.1999.00753.x 10321557

[B8] Krämer U Lemmen CH Behrendt H Link E Schäfer T Gostomzyk J Scherer G Ring J The effect of environmental tobacco smoke on eczema and allergic sensitization in children Br J Dermatol 2004 150 111 118 10.1111/j.1365-2133.2004.05710.x 14746624

[B9] Strachan DP Cook DG Health effects of passive smoking 5, Parental smoking and allergic sensitisation in children Thorax 1998 53 117 123 10.1136/thx.53.2.117 9624297 PMC1758719

[B10] Jaakkola JJ Nafstad P Magnus P Environmental Tobacco Smoke, Parental Atopy, and Childhood Asthma Environ Health Perspect 2001 109 579 582 10.2307/3455031 11445511 PMC1240339

[B11] Keil T Lau S Roll S Gruber C Nickel R Niggenmann B Wahn U Willich SN Kulig M Maternal smoking increases risk of allergic sensitization and wheezing only in children with allergic predisposition: longitudinal analysis from birth to 10 years Allergy 2009 64 445 451 10.1111/j.1398-9995.2008.01867.x 19170671

[B12] Raherison C Pénard-Morand C Moreau D Caillaud D Charpin D Kopferschmitt C Lavaud F Taytard A Maesano IA Smoking exposure and allergic sensitization in children according to maternal allergies Ann Allergy Asthma Immunol 2008 100 351 7 10.1016/S1081-1206(10)60598-4 18450121

[B13] Hagelin E Magnusson M Sundelin C Barnhälsovård 2007 4 Stockholm, Liber AB (In Swedish)

[B14] Rosvall M Falck S Moghaddassi M Köhler M Östergren P-O Rapport 2007: Barns hälsa och levnadsförhållanden Socialmedicinska Enheten, Malmö, Region Skåne 2007 (In Swedish)

[B15] Rothman KJ Modern Epidemiology 1986 Boston: Little Brown

[B16] Hosmer DW Lemeshow S Confidence interval estimation of interaction Epidemiology 1992 3 452 6 10.1097/00001648-199209000-00012 1391139

[B17] BAMSE projektet går vidare Vårdal nytt Nr 1 2003 http://vardal.orbelon.com/vnytt/1-03/art06.html (In Swedish)

[B18] Rothman KJ Greenland S Walker AM Concepts of interaction Am J Epidemiol 1980 112 467 70 7424895 10.1093/oxfordjournals.aje.a113015

[B19] Hallqvist J Ahlbom A Diderichsen F Reuterwall C How to evaluate interaction between causes: a review of practices in cardiovascular epidemiology J Intern Med 1996 239 377 82 10.1046/j.1365-2796.1996.431782000.x 8642229

[B20] Skrondal A Interaction as Departure from Additivity in Case-Control Studies: A Cautionary Note Am J Epidemiol 2003 158 251 58 10.1093/aje/kwg113 12882947

[B21] Dizier MH Bouzigon E Guilloud-Bataille M Siroux V Lemainque A Boland A Lathrop M Demenais F Evidence for gene x smoking exposure interaction in a genome-wide linkage screen of asthma and bronchial hyper responsiveness in EGEA families Eur J Hum Genet 2007 15 810 815 10.1038/sj.ejhg.5201830 17426724

[B22] Choudhry S Avila PC Nazario S Ung N Kho J Rodriguez-Santana JR Casal J Tsai HJ Torres A Ziv E Toscano M Sylvia JS Alioto ME Salazar M Gomez I Fagan JK Salas J Lilly C Matallana H Castro RA Selman M Weiss ST Ford JG Drazen JM Rodriguez-Cintron W Chapela R Silverman EK Burchard EG CD14 tobacco gene-environment interaction modifies asthma severity and immunoglobulin E levels in Latinos with asthma Am J Respir Crit Care Med 2005 172 173 182 10.1164/rccm.200409-1232OC 15879416

[B23] Colilla S Nicolae D Pluzhnikov A Blumenthal MN Beaty TH Bleecker ER Lange EM Rich SS Meyers DA Ober C Cox NJ Evidence for gene-environment interactions in a linkage study of asthma and smoking exposure J Allergy Clin Immunol 2003 111 840 846 10.1067/mai.2003.170 12704367

[B24] Kabesch M Gene by environment interactions and the development of asthma and allergy Toxicol Lett 2006 162 43 48 10.1016/j.toxlet.2005.10.009 16439069

[B25] Carlsen KH Carlsen KC Respiratory effects of tobacco smoking on infants and young children Paediatr Respir Rev 2008 9 11 19 10.1016/j.prrv.2007.11.007 18280975

[B26] The Swedish National Institute of Public Health http://www.fhi.se/sv/Handbocker/Uppslagsverk-barn-och-unga/Tobaksbruk---foraldrar (In Swedish)

[B27] Kummeling I Thijs C Stelma F Huber M Brandt PA Dagnelie PC Do parents with an atopic family history adopt a "prudent" lifestyle for their infant? (KOALA study) Clin Exp Allergy 2006 36 489 494 10.1111/j.1365-2222.2006.02473.x 16630154

[B28] Gorber SC Schofield-Hurwitz S Hardt J Levasseur G Tremblay M The accuracy of selfreported smoking. A systematic review of the relationship between self reported and cotinine assessed smoking status Nicotine Tob Res 2009 11 12 24 10.1093/ntr/ntn010 19246437

[B29] Nafstad P Kongerud J Botten G Urdal P Silsand T Pedersen BS Jaakkola JJ Fetal exposure to tobacco smoke products: a comparison between self-reported maternal smoking and concentrations of cotinine and thiocyanate in cord serum Acta Ostet Gynecol Scand 1996 75 902 907 10.3109/00016349609055025 9003090

[B30] Christensen AE Tobiassen M Jensen TK Wielandt H Bakketeig L Høst A Repeated validation of parental self-reported smoking during pregnancy and infancy: a prospective cohort study of infants at high risk for allergy development Paediatr Perinat Epidemiol 2004 18 73 79 10.1111/j.1365-3016.2003.00520.x 14738549

[B31] Torén K Palmqvist M Löwhagena O Balder B Tunsäter A Self-reported asthma was biased in relation to disease severity while reported year of asthma onset was accurate J Clin Epidemiol 2006 59 90 93 10.1016/j.jclinepi.2005.03.019 16360566

